# Recall of substandard medicines in Brazil during the period 2010–2018

**DOI:** 10.1186/s12913-023-09225-w

**Published:** 2023-03-10

**Authors:** Cristiani Lopes Capistrano Gonçalves Oliveira, Virgínia Freire Machado, Heitor de Freitas Tavares, Gabriel Lucas Marques Ribeiro, Paulo Sérgio Dourado Arrais

**Affiliations:** 1 Pharmacy Department, Faculty of Pharmacy, Dentistry and Nursing, Federal University of Ceará, Rua Pastor Samuel Munguba, 1210, Rodolfo Teófilo, Fortaleza, 60430-372 Ceará Brazil; 2 Lato Sensu post-graduation in pharmaceutical industry, Pharmacy Department, Faculty of Pharmacy, Dentistry and Nursing, Federal University of Ceará, Rua Pastor Samuel Munguba, 1210, Rodoldo Teófilo, Fortaleza, Ceará Brazil; 3 Pharmacy undergraduate student, Faculty of Pharmacy, Dentistry and Nursing, Federal University of Ceará, Rua Pastor Samuel Munguba, 1210, Rodolfo Teófilo, Fortaleza, Ceará Brazil

**Keywords:** ANVISA, Brazil, Generic medicine, GMP, Quality control, Recall, Reference medicine, Similar medicine, Substandard medicines

## Abstract

**Background:**

Even with all the care taken during the production process, the pharmaceutical industries are still subject to manufacturing medicines with quality deviations, generating commercialized products without the required quality and necessitating their subsequent recall from the market. The objective of this study was to evaluate the reasons that led to the recall of medicines in Brazil in the period evaluated.

**Methods:**

This is a descriptive study (using document analysis), on the recall of substandard medicines registered on the website of the National Health Surveillance Agency (ANVISA), from 2010 to 2018. The variables studied were the type of medicine (reference, generic, similar, specific, biological, herbal, simplified notification, new and radiopharmaceutical), type of pharmaceutical dosage form (solid, liquid, semi-solid and parenteral preparation), and reason for recall (Good manufacturing practices, quality and quality/good manufacturing practices).

**Results:**

A total of *n* = 3,056 recalls of substandard medicine were recorded. Similar medicines had a higher recall index (30.1%), followed by generics (21.3%), simplified notification (20.7%) and reference (12.2%). Different dosage forms had similar recalls: solids (35.2%), liquids (31.2%) and parenteral preparations (30.0%), with the exception of semi-solids (3.4%). The reasons for the highest occurrences were related to good manufacturing practices (58.4%) and quality (40.4%).

**Conclusion:**

The probable cause for this high number of recalls is the fact that, even with all the quality controls and processes in accordance with good manufacturing practices, errors can occur, both human and in automated processes, thus causing the release of batches that should not have been approved. In summary, it is necessary for manufacturers to implement a robust and well structured quality system in order to avoid such deviations, and it is up to ANVISA to apply greater oversight in the post marketing of these products.

## Background

Medicines that are unregistered or unlicensed by the health authority, as well as falsified, stolen, and smuggled medicines, along with those with quality deviations in their manufacturing process are a serious public health problem worldwide. This is especially in developing countries, because they present a risk to people's health as they do not offer the guarantees of effectiveness, safety and quality that are legally required [1–5].

Medicines with quality deviations (substandard medicines) are those that do not meet the requirements of the guidance of good manufacturing practices and, according to the WHO, are drugs manufactured outside of specifications, which do not meet the established quality criteria in the country where the drug is marketed [6–10].

In Brazil, it is the role of the Brazilian Health Regulatory Agency – ANVISA to supply product market surveillance with the objective of identifying possible substandard medicines and implementing actions that prevent or minimize health risks. Created in 1999, ANVISA is responsible for defining the criteria and steps necessary for clinical research (pre market), for the release of a new drug (registered medicine) and monitoring the product in the post market through the pharmacovigilance system. The registered medicine is only approved if the drug has proven quality, safety, efficacy and the company presents certifications of compliance of good manufacturing practice (GMP) and control of the location where the drugs will be produced [11].

In Brazil, a specific regulation determines that the pharmaceutical industry has to inform ANVISA and consumers about the recall of substandard medicines that present a health risk. In this regulation, the quality deviations are classified depending on the severity and risk to the consumer's health using the classifications Class I, II and III (Table 1) [12]. According to this regulation, the industry has the obligation to inform ANVISA if a drug has sufficient evidence or proof of quality deviation, and if the drug is classified as risk classification I and II. Failure to comply with the determinations of this regulation will subject the violator to legal punishments.

**Table 1:** Classification of quality deviations according to ANVISA regulation

Classification	Definition
Class I	Situation in which there is a high probability that the use or exposure to a drug could cause a health risk resulting in death, threat to life or permanent harm
Class II	Situation in which there is a high probability that the use of or exposure to a drug may cause temporary harm to health or that may be reversible by drug treatment
Class III	Situation in which there is a low probability that the use or exposure to a drug could cause adverse health consequences

The recall of medicinal products must also involve the entire chain of pharmaceutical products, covering the stages of production, distribution, transport and dispensing. The company's management system must ensure that it can track 100% of the units produced throughout the distribution chain, proving its effectiveness periodically through tests and evaluations with simulated recalls [13].

Drug recall in Brazil is defined as an action aimed at the immediate and effective withdrawal from the market of a certain batch(es) of drug, with sufficient evidence or proof of quality deviation, which may pose a risk to health, or upon cancellation of its registration. The safety and efficacy of the product is the responsibility of the company that holds the registration of the drug, and its distributors. Medications with quality deviation are an inevitable consequence of the pharmaceutical industry's failure to comply with good manufacturing practices [14, 15].

The WHO estimates that 10.5% of medical products marketed in developing countries have not met quality standards and good manufacturing practices [16]. In a recent study, the nonconformities of the good manufacturing practices of the Brazilian pharmaceutical industry were evaluated for three years (2015–2017). In this period, there were 485 inspections, with 61.4% of the inspected Brazilian companies considered satisfactory, 23.3% were “on hold” and 15.3% of the inspections were considered unsatisfactory. The most common areas of disability were qualification (Documented evidence that premises, systems or equipment are able to achieve the predetermined specifications when properly installed and/or working correctly and lead to the expected results) and validation (Action of proving and documenting that any process, procedure or method actually and consistently leads to the expected results), documentation and facilities. The number of companies that present critical content was around 74 companies, a relatively high number when compared to other countries, and directly reflecting on the high recall of medicines in the Brazilian market [17].

ANVISA, in a 2-year period (2015–2016), conducted 255 international inspections in 41 countries. In the period analyzed 62.7% of the companies inspected by ANVISA were classified as satisfactory, 24.7% received on-demand (under assessment) status, and 12.5% of the inspections concluded that the companies did not meet GMP (unsatisfactory). Most of the deficiencies found were considered minor (57.2%), but many major deficiencies (40.3%) were also found. Critical deficiencies were a small part of the total (2.5%) [18]. Deficiencies are classified on a case-by-case basis, but in regard to critical deficiencies we can cite as examples the production of medicine using raw materials/processes different from those registered in ANVISA; improper sharing of production lines; and evidence of widespread cross-contamination, among others. In regard to major deficiencies, we can give as examples: failure to perform tests on the raw material received, absence of physical–chemical and microbiological tests on water for pharmaceutical use, and absence of sampling in each sterilization load to perform the sterility test, among others. Additionally, in regard to minor deficiencies, we can mention the inadequate storage of medicines, storage of documents for insufficient time, and unidentified packaging lines in accordance with the product being packaged, among others [19].

In Brazil, between 2012 and 2017, notifications of irregular products (any products that did not meet the rules defined by ANVISA, for example, medicines, active pharmaceutical ingredient, cosmetics, health products, sanitizers, blood, blood components) in the market were analyzed and showed that 38.5% of notifications were of medicines. In this article, 807 measures indicated by ANVISA for the recall of medicines were analyzed, with 1,149 drugs and 254 companies involved, and it was observed that 55.9% were of medicines that were found to have quality deviation. The most frequent therapeutic classes of irregular drugs were antibacterials, analgesics and antivirals with 13.2%, and herbal products led the list of those unregistered or from companies presenting an irregular situation (non-compliance with regulations, company without operating permit and/or certificate of Good Practices; general changes) [20]. The problem is not only restricted to Brazil, because the situation is present in both developing [21–25] and developed countries [26–30].

In Brazil, there are records of serious cases of substandard medicines that caused the death of several people. In 2003, the drug celobar®, barium sulfate, used as a contrast agent in medical examinations, was marketed with a high percentage of barium carbonate (highly toxic) due to the synthesis performed in the laboratory itself to obtain barium sulfate. This case caused the death of more than 20 people [31].

In the literature, there are also reports of commercialized antimicrobials around the world that present quality deviations, mainly in relation to the active ingredient content, which can contribute to the development of bacterial resistance and increased morbidity and mortality [32–34].

The marketing of substandard medicines is a persistent problem in some countries and exposes patients to the use of drugs that may not be effective or safe. Until now, there was no study in the literature on medicine recall in Brazil thus this is an important study for the pharmaceutical industry and ANVISA itself. [35].

Given the above, this study aims to identify and evaluate the reasons that led to the recall of drugs with quality deviation from the Brazilian pharmaceutical market in the period from 2010 to 2018, and which were registered in ANVISA's inspection and monitoring system.

## Methods

### Type of study/inclusion and exclusion criteria

This is a descriptive study (documentary analysis) which is retrospective and quantitative on the recall of medicines with quality deviation, registered in ANVISA's inspection and monitoring system, located on their website, from 2010 to 2018 [36].

The period of this study was chosen according to the validity of the guidelines for GMP, which were in force between 2010 and 2018. In 2019, a new set of guidelines for good manufacturing practices in Brazil was published, reflecting the entry of ANVISA into the ICH, and demonstrated their need to harmonize internal regulations with the ICH guidelines.

In this study, only medicines that had quality deviations in the manufacturing processes, unregistered/unlicensed medicines, medicines that had problems with good manufacturing practices, or that generated product recall as a preventive measure were considered (medicines that are recalled, but quality assessment is necessary after recall). Cases where industries did not have authorization to manufacture drugs and that the drugs were falsified [10] were excluded.

The data on drugs with quality deviation were obtained through the ANVISA website, using the link “subjects”/ "inspection and monitoring" / "irregular products". The file made available on the ANVISA website lists the drugs recalled in a table, where the following data are available: company name, product, lot /validity, inspection action, reason for recall, and publication in the official government journal. In the case of incomplete information or where doubts were generated, the publication in the official government journal was consulted and the data supplemented [37].

In the data collection, the drugs and the respective and different batches were counted and reported, even if it was the same drug but with different batches. When the available file read "recall of all batches on the market", this was classified as a single batch.

### Classification according to the type of medicine and dosage form

The medicines were classified as generic, similar, reference, new [11], herbal, biological, specific [38], radiopharmaceutical and simplified notification [39] (Table 2). Generic, similar and reference medicines were listed through an active search on the ANVISA website (lists A and B of reference medicines related to each year of study) and ANVISA's list of generics and similars. Regarding the dosage form, the medicines were divided into: solid dosage form (tablets, hard and soft capsules, pillules (sugar coated tablet*)*, coated granules in sachet, powder for oral solution, vaginal tablets, suppositories, among others), liquid (oral solution, topical solution, syrup, suspension, among others), semi-solid (cream, ointment, lotion, gel, among others) and parenteral preparation (solution for injection, lyophilized powder for solution for injection, suspension for injection, solution for peritoneal dialysis, among others).

**Table 2:** Definition of types of medicines

Type of medicine	Definition
Reference	An innovative product registered with ANVISA and marketed in the country, whose effectiveness, safety and quality were scientifically proven by the competent federal agency at the time of register
Generic	A generic drug is defined as a drug with the same active pharmaceutical ingredient (API), dosage form, safety, quality, and efficacy as the original proprietary drug, with which it can be interchangeable
Similar	Medicine that contains the same API, dosage form, strength, indication, and posology of the original proprietary drug but is identified by a brand name, with which it can be interchangeable with reference medicine
Specific	Specific medicines are pharmaceutical products, technically obtained or prepared, with a prophylactic, curative or palliative purpose that do not fall into the categories of new, generic, similar, biological, herbal or notified medicine and whose active substance(s), regardless of nature or origin, is not subject to bioequivalence testing against a comparable product
New	These are innovative medicines that are registered in the country for the first time. Generally, its active principles are new, synthetic or semi-synthetic molecules, associated or not with other active principles
Herbal	These are obtained with the exclusive use of active vegetable raw materials
Biological	Biological medicines are complex molecules of high molecular weight obtained from biological fluids, tissues of animal origin or biotechnological procedures through manipulation or insertion of other genetic material (recombinant DNA technology) or alteration of genes that occurs due to irradiation, chemicals or forced selection
Radiopharmaceutical	Pharmaceutical preparations for therapeutic purposes which, when ready for use, contain one or more radionuclides
Simplified notification	They are low-risk drugs for which companies do not need to submit documentation and wait for the register to be evaluated and published; Companies only inform ANVISA that they are producing the drug, which must be on the list of low-risk drugs subject to simplified notification (E.g. sodium chloride solution, aluminum hydroxide suspension, folic acid solution, and chlorhexidine hydrochloride solution)

### Classification in regard to the general and specific reason for recall

The medicines recalled were classified and quantified for general and specific reasons (Table 3). The specific reasons were established according to the provisions of good manufacturing practices and the Brazilian Pharmacopoeia.

**Table 3:** Classification in regard to the general and specific reason for the recall of medicines with quality deviation

General reason	Specific reason
Good Manufacturing Practices (GMP)	(1) Irregularities in health standards; (2) Exchange of packaging/labels/package insert during production; (3) Unlicensed/unregistered medicines; (4) Nonconformities with registered medicines; (5) Not specified**;**
Quality	(1) Appearance (2) Package insert (3) Microbiological control (4) Weight determination (5) pH determination (6) Volume determination (7) Disintegration test (8) Dissolution test (9) Hardness (10) Packaging (11) Bacterial endotoxin (12) Limit test (13) Potency test (14) Stability study (15) Less active polymorphic forms (16) Friability (17) Impurity (18) Identification of ethyl alcohol (19) Unspecified (20) Visible particles (21) Uniformity of dosage units (22) Labeling (23) Content (24) Donor blood detected with sporadic Creutzfeldt-Jakob diagnosis (25) Sterility test (26) Injectability test (27) Molecular distribution test
Not specified	Not specified
Good Manufacturing Practices/ Quality	Involving GMP and Quality

For the variable “Good Manufacturing Practices”, the specific reason “nonconformities with registered medicines” was related to the following reasons: “Change of name/excipient/place of manufacture/packaging/expiry date/method of manufacture”, among others, which were registered in ANVISA with unauthorized modification.

In the “quality” item, the specific reasons were also related to the recall for more than one specific reason, for example: Appearance and content, dissolution and content, labeling and packaging, among others.

The data were stored in an Excel program file and analyzed by the statistical tools of the program, with absolute and relative frequencies being presented.

## Results

### Type of medicine and dosage form

In the period of the study a total of 3,056 medicine recalls were identified (Table 4). The highest number of recalls occurred in 2013 (*n* = 443; 14.5%) and 2017 (*n* = 845; 27.7%), caused in the first case by the recall of 176 drugs from the same pharmaceutical industry, and in the second case by the recall of 514 batches of simplified notification medicines (chlorhexidine hydrochloride solution, *n* = 487) in different concentrations, supplied by the same laboratory.

**Table 4:** Types of medicines recall between 2010 and 2018

Medicine	Year									Total of medicine recalls n (%)
	2010	2011	2012	2013	2014	2015	2016	2017	2018	
Reference	18	18	27	62	27	25	85	51	59	372 (12.2)
Similar	94	71	239	58	160	83	58	136	21	920 (30.1)
Generic	37	24	65	250	53	55	45	72	48	649 (21.3)
Simplified notification	7	9	8	40	14	11	13	514	17	633 (20.7)
specific	14	14	19	9	20	83	12	59	12	242 (7.9)
Biological	19	9	13	11	16	29	6	1	0	104 (3.4)
Herbal	8	3	6	13	4	37	15	6	0	92 (3.0)
New	0	1	0	0	0	1	15	5	13	35 (1.1)
Radiopharmaceutical	0	1	0	0	0	0	1	0	0	2 (0.1)
Not specified	0	0	0	0	0	1	3	1	2	7 (0.2)
Total of recall per year. n. (%)	197 (6.5)	150 (4.9)	377 (12.3)	443 (14.5)	294 (9.6)	325 (10.6)	253 (8.3)	845 (27.7)	172 (5.6)	3,056

In 2016 (*n* = 253), there was a high number of reference medicine recalls, caused in this case by the recall of 59 batches of indapamide prolonged-release tablets due to a change in formulation, without the prior consent of ANVISA (nonconformities with registered medicines). In relation to a similar medicine, in 2012 there was a greater number of recalls (*n* = 239) due to the suspension of a line of solids from a pharmaceutical industry resulting in the recall of 184 batches of different medicines. In 2013 there was a greater amount of recalls of generic medicines (*n* = 250) due to there being two large recalls: 54 batches of the injectable solution of imipenem and cilastatin, due to problems in the labeling, and 176 batches of injectable solution of dipyrone, due to irregularities in good manufacturing practices. In general, the medicine recall involves mainly similar (30.1%), generic (21.3%) simplified notification products (20.7%) and reference medicines (12.2%) (Table 4).

**Fig. 1:**
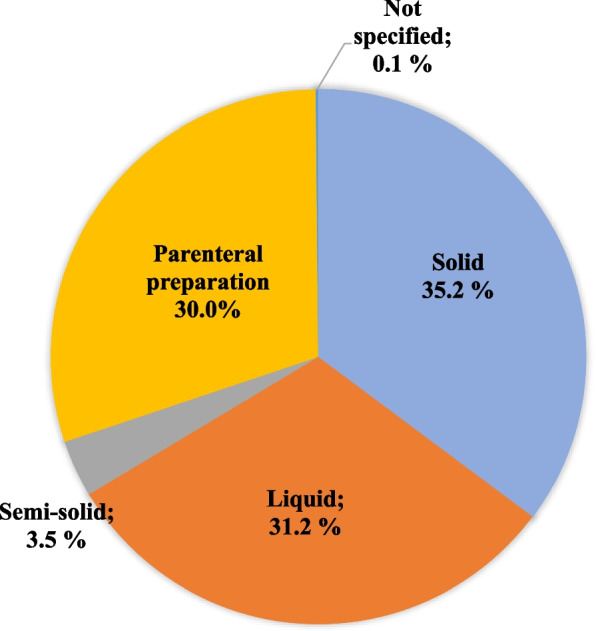
Dosage forms recalled during the period from 2010 to 2018 (*n* = 3,056)

Table 5 shows the reasons for recall according to drug type. GMP was the main reason for the recall of these medicines with the exception of reference medicine (49.7%), specific (80.6%) and biological (68.3%), where quality was the main reason for the recall. Regarding the type of dosage form (Fig. 1), the solid dosage form (*n* = 1076) obtained 35.2% of recall, where tablets obtained the highest number of recalls (*n* = 824, 76.6%), followed by hard capsules (*n* = 83, 7.7%), pillules (*n* = 67, 6.2%) and others (*n* = 102, 9.5%).

**Table 5:** Type of medicine and reason for the recall

	Similar n (%)	Generic n (%)	Reference n (%)	Simplified notification n (%)	Specific n (%)	Biological n (%)	Herbal n (%)	New n (%)	Not specified n (%)	Radiopharmaceutical n (%)
GMP	521 (56.6)	406 (62.5)	155 (41.7)	515 (81.4)	47 (19.4)	30 (28.8)	79 (85.9)	23 (65.7)	7 (100)	1 (50)
Quality	398 (43.3)	241 (37.1)	185 (49.7)	118 (18.6)	195 (80.6)	71 (68.3)	13 (14.1)	12 (34.3)	0	1 (50)
Quality/GMP	0	1 (0.2)	29 (7.8)	0	0	0	0	0	0	0
Not specified	1 (0.1)	1 (0.2)	3 (0.8)	0	0	3 (2.9)	0	0	0	0
Total (n)	920	649	372	633	242	104	92	35	7	2

The recalls with the highest impact in the liquid dosage form (*n* = 954) were those with topical solution (*n* = 547, 57.3%), followed by oral suspension (*n* = 182, 19.1%), oral solution (*n* = 99, 10.4%) and others (*n* = 126, 13.2%). Parenteral preparations constituted 30.0% (*n* = 917) of recall, where njectable solutions correspond to 64.1% (*n* = 588), powder for solution for injection 19.1% (*n* = 175), solution for peritoneal dialysis 7.8% (*n* = 71), and others 9.0% (*n* = 83). The semi-solid dosage form (*n* = 105) had a few recalls, with emphasis on lotion (*n* = 24, 22.9%), ophthalmic ointment (*n* = 23, 21.9%), cream gel (*n* = 15, 14.3%), followed by others (*n* = 43, 40.9%).

### Reason of recall

**Fig. 2:**
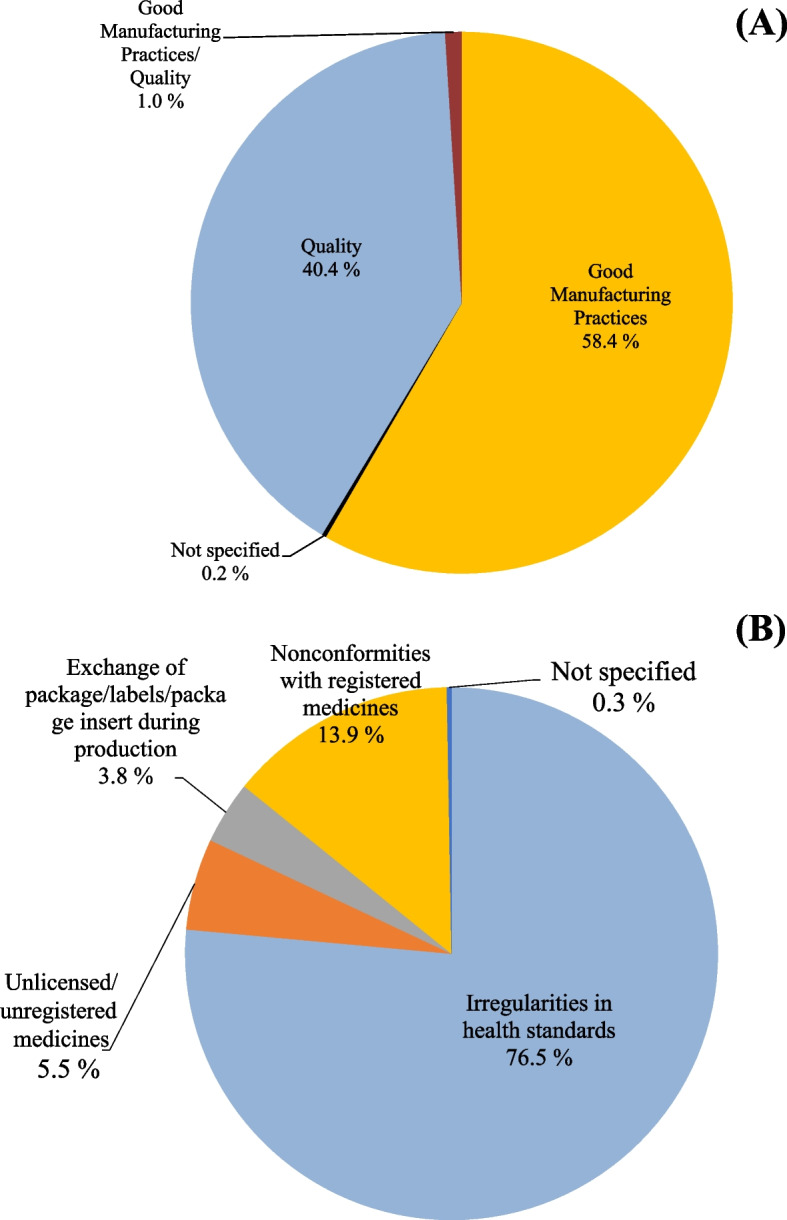
**A** Reason for drug recalls extracted from the ANVISA website (*n* = 3,056) and (**B**) classification of the reasons for GMP-related recalls (*n* = 1,784)

The general reasons that caused the recalls of medicine during the investigated period (2010–2018) were problems related to Good Manufacturing Practices (58.4%), followed by quality (40.4%) (Fig. 2A). The main specific reasons related to Good Manufacturing Practices were irregularities in health standards (76.5%) (Fig. 2B).

When assessing the reasons for recall per year, there was a greater variation in relation to good manufacturing practices: 2018 (*n* = 172, 55.8%), 2017 (*n* = 845, 80.4%), 2016 (*n* = 253, 65.2%), 2013 (*n* = 443, 50.1%), 2012 (*n* = 377, 72.1%); and quality: 2015 (*n* = 325, 61.8%), 2014 (*n* = 294, 62.9%), 2011 (*n* = 150, 59.3%) and 2010 (*n* = 197, 69.5%).

**Fig. 3:**
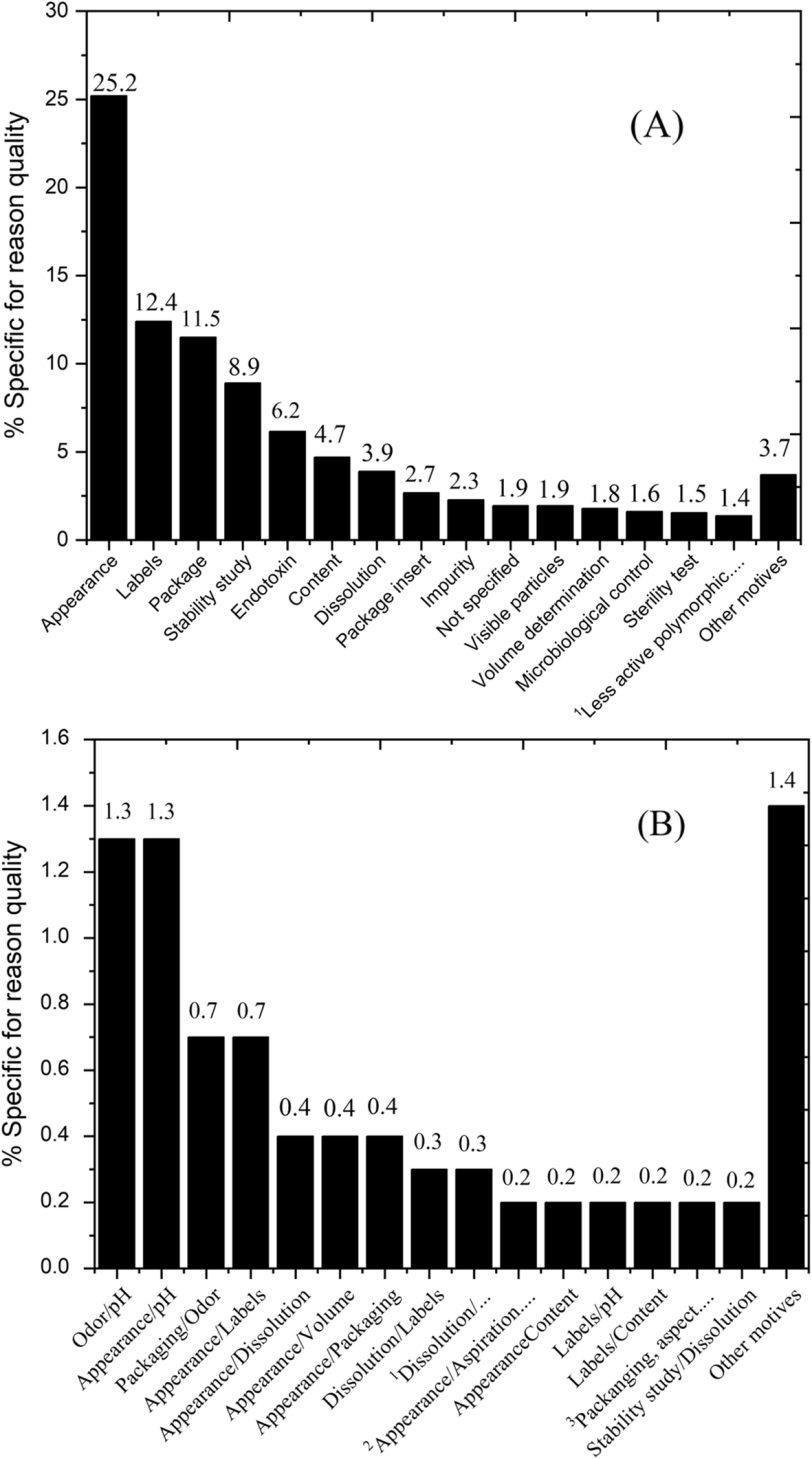
**A** Classification of quality-related recalls reasons and (**B**) Recalls related to more than one reason in the quality parameter (*n* = 1,234). Note: (A)^1^Less active polymorphics forms (Changes in the crystalline arrangement of a substance, without observing changes in the structure of molecules). (B)^1^Dissolution/Weight variation; ^2^Appearance/Aspiration and extrusion test; ^3^Packaging, aspect and count of microorganisms

Regarding the general quality reason (*n* = 1,234), 91.6% corresponded to recall for a specific reason and 8.4% for more than one specific reason. Figure 3(A) shows the main specific reason for recall in relation to quality, where appearance (25.2%), labels (12.4%) and packaging (11.5%) were the most quality problems identified. Other reasons represented less than 1.0% of the recall, considering each test individually: pH determination (0.6%), molecular distribution (0.6%), unit dose uniformity (0.5%), related substance (0.5%), aspiration and extrusion test (0.3%), weight determination (0.2%), disintegration test (0.2%), hardness (0.2%), donor blood detected with diagnosis of Sporadic Creutzfeldt-Jakob (0.2%), friability (0.1%), Iron II limit test (0.1%), potency (0.1%) and identification of ethyl alcohol (0.1%).

Other recalls involved more than one specific reason (Fig. 3B), in which odor/pH and appearance/pH represented 1.4% of the recall lots. The following situations were identified with less than 0.1% incidence: appearance and friability; Disulfide content and limit; appearance and count of total microorganisms; Labeling, identification of markers, loss on drying, content; Assay and uniformity of unit dose; Volume and secondary packaging; Label/packaging; Labeling, content and identification; Label and package insert; appearance, Labeling analysis and packaging appearance; appearance, color and volume; appearance, pH, content and label; Content and drip test; and appearance / determination of volume / content.

## Discussion

This study reports on the features of recalled medicines in Brazil in the nine years investigated. During this period, similar medicines were the type that were most recalled, followed by the solid dosage form, while the reason for recall was most commonly related to good manufacturing practices and quality. Among the GMP issues, the one that stands out most was “irregularities in health standards” and in regard to “quality” issues the problems were in appearance.

It was observed that in the ANVISA table there was no classification (class I, II and III) of the quality deviations that caused the recall until the last analyzed year (2018). This was also observed in a recent article, which stated that the risk classification was not included because ANVISA's documentation did not have this information [20]. This type of classification is very important to assess the health risk of the population exposed to a substandard medicine on the market, because class I drugs involve the risk of harm to health or even death. In the forms to be filled out in the recall action this information is mandatory, but it is emphasized here that the table provided by ANVISA does not report the type of health risk classification for the population in regard to the recalled drugs.

### Type of medicine and dosage form

Before the establishment of ANVISA in 1999, similar medicines registered in Brazil were not required to carry out pharmaceutical equivalence and relative bioavailability tests. In 2003, ANVISA published specific regulations informing about the mandatory performance of equivalence and bioavailability tests, giving a period of 10 years for certain medicines. In 2014, with the period for presentation of the tests at the time of registration coming to an end, ANVISA published RDC 58/2014, which detailed all similar medicines that had already proved their pharmaceutical equivalence and relative bioavailability [40].

Reference medicines had a high frequency of recall between 2010 and 2018. This type of medicine is considered an innovative, branded medicine used by generics and similar for future registrations of medicines that are candidates for commercialization, since in pharmaceutical equivalence and bioequivalence / relative bioavailability studies, generic and similar medicines need to be compared to the reference. It is of upmost importance that these medicines maintain a constant quality and RDC No. 35/2012, deals with the criteria for inclusion and exclusion of reference medicines in Brazil [41].

Simplified notification drugs had a high recall rate. This type of product must follow the monographs of the Brazilian pharmacopoeia or other compendium recognized by ANVISA to generate the quality control analysis reports of the finished product. The fact that we have multiple recalls may be indicative of non-compliance within these rules. The renewal of authorization of this type of product occurs every five years and these results may impair its renewal [39].

Regarding the dosage form, equivalent recalls were obtained for solid, liquid and parenteral preparation, the latter being of concern because it can cause serious damage to the patient due to its direct availability in the bloodstream.

### Reason for medicine recall

Regarding the general reasons for recall, good manufacturing practices and quality were the parameters that caused a greater number of recalls. When assessing the reasons for recall per year, it is found that there is a very high variation and fluctuation, with quality drawing a lot of attention, as there are lots of products on the market that are released outside of specifications.

### Good manufacturing practices

When evaluating the specific reasons within the “good manufacturing practices”, the item “irregularities in the health standards”, which involves irregularities during the inspection of ANVISA and not meeting the requirements of good manufacturing practices, represents the main cause of recall, and is directly linked to the GMP regulation in Brazil and its listed requirements.

A worrying statistic is the “nonconformities with registered medicine”. Any medication to be marketed needs to be registered with the entire documented production process, this includes batch size, excipients used, among other aspects. This implies that during the period of authorization of a registered medicine the industry must follow all documents submitted to ANVISA (validation processes, stability studies, qualified suppliers, among others). Among the nonconformities, "alteration in the mode of manufacture" and "alteration of formulation" are points of concern, as they can directly affect the bioavailability of the drug, making it possible to use the medicine without any therapeutic effect. For the medicines recalled in relation to "change in the method of manufacture" or "formulation", in most cases ANVISA determine the recall of "All batches". During the period of this study, it was verified that in 2016 there was the recall of three different reference medicines due to the following nonconformities: change in the API synthesis route, change in the product composition formula, moderate change in configuration of excipient, and change in the expiration date of the drug from 24 to 36 months. These changes differed with what was approved in the initial registration of the medicine and all of them were made without prior consent from ANVISA. In 2015, for example, a specific medicine (epinephrine nasal solution) changed the API manufacturer without prior authorization from ANVISA.

Another relevant point is the large number of recalls due to a lack of “product registered”, thus showing that ANVISA is capable to identify and remove these illegal products from the market and shows that many manufacturers do not respect the current legislation in regard to producing irregular medicines and exposing the population to risks with their life and health. In this way, warning campaigns in the media could be promoted, in order to encourage the population to always check if the medication being purchased is registered with ANVISA, because currently we have seen few actions in this regard.

The aspect that involves changing packaging/label and changing the package insert are reasons that can bring great risks to the consumer's health. During this period, 49 batches were recalled due to changing the packaging/label and 19 batches due to changing the package insert. Of the recalled drugs, there were morphine tablets, a reference medicine, which were recalled due to the use of product cartridges with a concentration of 10 mg being sold in the packaging of morphine of 30 mg, demonstrating flaws in good manufacturing practices.

### Quality

In relation to the general reason for quality, which is defined as the nonconformity of the quality parameters established for a product according to Brazilian Pharmacopoeia specifications, appearance showed a high occurrence of nonconformities. Even with all the process controls, according to GMP, there can be both human error as well as failures in automated processes that cause the release of batches that should have been disapproved and, consequently, not released for consumption.

Problems with labeling and packaging presented a considerable percentage in the recall of products, as these are used to protect medicines and identify them properly. This demonstrates to the manufacturer that packaging and labels must also follow all error and risk analysis standards, which are sometimes just limited to the product itself. Recall due to the presence of bacterial endotoxin represents a high risk to the consumer and releasing contaminated batches demonstrates a serious failure in the quality control of the industry. Incorrect dissolution is a problem and limits the possible therapeutic effect of the drug, because the dissolution is a fundamental step for the complete and correct absorption of the drug that was administered.

In the quality parameter, there were recalls that showed nonconformity in more than one quality test, and again, there is the question of appearance in this regard, involving appearance and pH, odor and pH, and packaging and odor, however, it was appearance that presented a greater number of recalls. The recall for more than one specific reason, related to quality, is a concern, because the industry has supplied batches with considerable quality deviations, including quality assurance and quality control, for commercialization.

### Good manufacturing practices and quality

The reasons for recall related to the two variables, good manufacturing practices and quality, represented a high frequency of the recalled batches. These recalls occurred in 2013 in which a reference medicine (Imipramine hydrochloride) was recalled for presenting results above the specification values for the degradation product G22358 (Quality), as well as presenting an expiration date not authorized by ANVISA.

### Recommendations

This type of study is important in order to improve ANVISA assessment guidelines, because they identify the types of medicines recalled, the reasons and causes involved, and they can alert companies to this problem. As a regulatory agency established just over 20 years ago, its internal guidelines are constantly changing in order to improve the production and quality control of medicines in the country, seeking to guarantee the effectiveness and safety of medicines. In 2019, a new set of guidelines for good manufacturing practices in Brazil was published, reflecting the entry of ANVISA into the ICH, and demonstrated their need to harmonize internal regulatory with the ICH guidelines. This study, involving the survey of medicine recalls in Brazil in the period before entering the ICH, will be an important reference to evaluate the benefits of aligning the internal rules in accordance with the ICH documents, seeking to improve regulatory issues in the country and the regulatory convergence with other countries.

The recall of medicine becomes compulsory when errors were observed in terms of quality or good manufacturing practices. The medicine recall process is costly for the pharmaceutical industries, as the manufacturer spent money on production, had costs to place the product on the market, both with distribution and advertising, but also had costs throughout the distribution chain, and thus will have logistical and reimbursement costs for distributors and even consumers.

The industry must invest in training employees and motivate them to comply with the procedures described for the production processes, thus avoiding changes in the manufacturing process however small, so that they do not affect the quality of the product.

Manufacturers should always seek the use of mechanisms that minimize the possibility of errors and dependence on the action and attention of employees to avoid these errors. To achieve this, there has been strong investment by manufacturers in the implementation of computerized systems in pharmaceutical processes and equipment, which continuously check for possible inconsistencies and deviations and determine process parameters, significantly reducing the possibility of errors.

As an example, there is an action that has contributed considerably to the safety of the processes, which is the installation of sensors in the packaging equipment and devices that evaluate 100% of the units produced, discarding any incorrect samples through cross contamination. It is also possible to carry out a risk analysis by production line and thus enable risk mitigation or elimination actions. In addition, it is possible to implement the presence of quality inspectors within the production area to assist in the identification of critical points and also in the anticipation of possible quality deviations. [42, 43].

To help solve these problems a closer relationship needs to be developed between ANVISA and the laboratories, with an exchange of information on regulatory requirements, with explanatory guides and periodic meetings/lectures in regard to the most frequent doubts. There should also be an increase in inspections in the laboratories in order to guarantee the effectiveness and safety of medicines marketed in Brazil.

### Limitations

 It can be seen from the data extracted from the ANVISA website that in the case of some recalls, the agency did not report the exact number of batches recalled and the information merely said, “recall all batches” (The use of this imprecise information was present in all years included in this study). This lack of information about the exact number of batches recalled may have resulted in the reporting of a smaller number of recalls than actually occurred during those years. Another limitation was that ANVISA did not report the class of risk of the collected drugs. This is important data for the consumer and is extremely relevant in statistical research on recalls.

## Conclusion

The recalls of substandard medicines were related mainly to good manufacturing practices and quality. Irregularities in health standards were the main reason for GMP recalls and appearance for quality recalls.

Even with all the requirements of GMP standards established by ANVISA for medicine manufacturers, there is still a large number of recalls due to deviation in quality and good manufacturing practices, showing that the production processes still produce errors in their stages, which enable the release of substandard medicines in the market, putting the population's health at risk.

## Data Availability

The datasets used and/or analyzed during the current study is from the corresponding author on reasonable request.

## Abbreviations

ANVISA: Brazilian Health Regulatory Agency
GMP: Good Manufacturing Practices
RDC: Collegiate Directorate Regulation
US: United States
WHO: World Health Organization
